# Supplementary material for: Effects of cannabis ingestion on endometriosis-associated pelvic pain and related symptoms
Source: PLoS One. 2021 Oct 26;16(10):e0258940. doi: 10.1371/journal.pone.0258940 (PMC8547625; doi:10.1371/journal.pone.0258940)
Supplement: Attachment — Submitted filename: Response to Reviewers.docx [file pone.0258940.s001.docx]

| Reviewer comment | Author response |
| --- | --- |
| The study is well thought out and properly written. In the introduction you should add that endometriosis not only affects women of childbearing age, but also postmenopausal (cites "Retroperitoneal endometriosis in postmenopausal woman causing deep vein thrombosis: case report and review of the literature, Ianieri MM, Clin Exp Obstet Gynecol. 2017; 44 (1): 148-150)  The materials and methods are clear as well as the results. The discussion should also be improved by talking about the potential hypertonus pelvic muscles of patients with endometriosis (cite DOI 10.1002 / uog.18924, Mabrouk M 2018) and the potential effect of cannabis at therapeutic dosage. | We thank the reviewer on the positive feedback relating to our manuscript being clear, well thought out and properly written. Specific to the reviewer’s comments relating to the inclusion of recommended papers within our manuscript, we have reviewed the suggested papers and included both references, and subsequent narrative, within the introduction and discussion sections of the manuscript.  The following lines refer to the *Revised manuscript with Track Changes* document.  Please see Lines 63-64:  “*In addition, endometriosis can also affect post-menopausal women, ranging from between 2-5% of cases*.” [5]  With included reference [5] on lines 399-402:  Ianieri MM, Buca DIP, Panaccio P, Cieri M, Francomano F, Liberati M. Retroperitoneal endometriosis in postmenopausal woman causing deep vein thrombosis: case report and review of the literature. Clin Exp Obstet Gynecol. 2017;44(1):148-50. Epub 2017/01/01. PubMed PMID: 29714887.  Please see Lines 125-126:  this project was exempt from ethical review “*by the Western Sydney University Human Ethics Committee*”  Please see Lines 250-253:  “*This may also be relevant for patients with Deep Infiltrating Endometriosis (DIE) who suffer from pelvic floor muscle hypertonic dysfunction, [34] whereby the muscle relaxant activity associated with cannabis [35] may also play an important role*.”  With included reference 34 on lines 516-521:  Mabrouk M, Raimondo D, Del Forno S, Baruffini F, Arena A, Benfenati A, et al. Pelvic floor muscle assessment on three- and four-dimensional transperineal ultrasound in women with ovarian endometriosis with or without retroperitoneal infiltration: a step towards complete functional assessment. Ultrasound Obstet Gynecol. 2018;52(2):265-8. Epub 2017/10/13. doi: 10.1002/uog.18924. PubMed PMID: 29024196.  With included reference 35 on lines 522-524:  Pertwee RG. Neuropharmacology and therapeutic potential of cannabinoids. Addict Biol. 2000;5(1):37-46. Epub 2000/01/01. doi: 10.1080/13556210071252. PubMed PMID: 20575818. |
